# Knowledge, Attitudes, and Practices Regarding COVID-19 Among Health Care Workers in Public Health Facilities in Eastern Ethiopia: Cross-sectional Survey Study

**DOI:** 10.2196/26980

**Published:** 2021-10-01

**Authors:** Alinoor Mohamed Farah, Tahir Yousuf Nour, Muse Obsiye, Mowlid Akil Aden, Omar Moeline Ali, Muktar Arab Hussein, Abdullahi Bedel Budul, Muktar Omer, Fentabil Getnet

**Affiliations:** 1 Department of Public Health Nutrition School of Public Health, College of Medicine and Health Sciences Jigjiga University Jigjiga Ethiopia; 2 Department of Epidemiology and Biostatistics School of Public Health, College of Medicine and Health Sciences Jigjiga University Jigjiga Ethiopia

**Keywords:** COVID-19, knowledge, attitude, practice, health care workers, Eastern Ethiopia

## Abstract

**Background:**

On March 13, 2020, Ethiopia reported the first confirmed case of COVID-19 in Addis Ababa. COVID-19 is likely to overwhelm an already-fragile health care delivery system and reduce the availability of essential health services. This analysis of data from the Somali Region of Eastern Ethiopia on health care workers’ (HCWs) knowledge, attitudes, and practices regarding the prevention and control of COVID-19 may be used in planning health education programs about the emerging viral disease.

**Objective:**

This study aimed to investigate the knowledge, attitudes, and practices of HCWs regarding COVID-19 infection.

**Methods:**

This cross-sectional study was conducted among HCWs in three public health facilities in the Somali Region, Eastern Ethiopia. A self-administered questionnaire was shared with all HCWs working at the public health facilities. A total of 15 knowledge questions were scored as 1 or 0 for correct or incorrect responses, respectively. A total of 14 practice questions were scored on a 3-point scale from 1 (“always”) to 3 (“never”). A total of six attitude questions were rated on a 5-point Likert scale, in a negative dimension, as follows: 1 (“strongly agree”), 2 (“agree”), 3 (“neutral”), 4 (“disagree”), and 5 (“strongly disagree”). Mean scores were calculated and used as a cut point to dichotomize the outcome variables (>13.7 indicated good knowledge, <18.8 indicated good practices, and ≤10.5 indicated favorable attitudes). We used *t* tests and analyses of variance (ie, *F* tests) to analyze the mean score differences of knowledge, attitudes, and practices between the independent variables. Spearman correlation was used to assess the relationship between mean knowledge and attitude scores.

**Results:**

Of the 686 HCWs approached, a total of 434 HCWs responded (63.3% response rate). The mean age of the participants was 27.6 (SD 5.3) years, and the majority of the participants were male (293/434, 67.5%). The mean knowledge score was 13.7 (SD 2.6), and 73.3% (318/434) of participants had sufficient knowledge. The mean attitude score was 10.5 (SD 4.1), and 54.8% (238/434) of the participants had a good attitude toward COVID-19. The mean practice score was 18.8 (SD 5.8), and 61.5% (267/434) of the participants practiced precautionary measures to prevent COVID-19. There was a negative correlation between knowledge and attitude scores (*r*=–0.295, *P*<.001) and between knowledge and practice scores (*r*=–0.298, *P*<.001).

**Conclusions:**

The overall levels of knowledge and practice were relatively better than the attitude level. This highlights the need to implement strategies that enhance the positive attitudes and safe practices of the HCWs for better containment of the pandemic and supporting of essential health care services.

## Introduction

COVID-19 is an emerging, rapidly changing global health challenge affecting all sectors, including the health sector [1]. In Ethiopia and in the Somali Region, in particular, there is already a limited number of health care workers (HCWs) in the health sector, which puts extreme strain on the capacity to serve patients, especially for nonemergency care [2]. Thus, COVID-19 is likely to overwhelm an already-fragile health care delivery system and reduce the availability of services for endemic health concerns, such as malaria and diarrheal diseases including cholera [3]. The proportion of Ethiopia’s water supply that is safe is 30%, which means the water supply serves as the leading cause of communicable diseases in the country. As a result, pre-existing inadequate hygiene practices, insufficient coverage in water and sanitation services, and overcrowding living situations all contribute to the virus’s occurrence and transmission. The pandemic is also adding to the burden of endemic infectious diseases that prevail in many countries with an ongoing humanitarian response; these diseases include cholera, measles, malaria, HIV, and tuberculosis.

To date, no specific antiviral treatment has been confirmed to be effective against COVID-19 [4]. Regarding infected patients with COVID-19, it has been recommended to apply appropriate symptomatic treatment and supportive care [5,6]. Therefore, applying preventive measures to reduce the spread of disease is of the utmost importance.

In this regard, airborne precautions and other protective measures have been discussed and proposed for prevention. Recommended preventive measures include regular handwashing with soap, social and physical distancing, and respiratory hygiene (ie, covering the mouth and nose while coughing or sneezing) [5,7].

The World Health Organization (WHO) also issued guidelines on the use of face masks in different settings, including in the community, in home-based care, and in the health care settings of COVID-19 [8]. In this guideline, HCWs are recommended to use face masks, such as certified N95 respirator masks, when performing aerosol-generating procedures and to use medical masks when providing care to suspected or confirmed COVID-19 cases. However, these effective prevention and control practices depend on awareness and compliance among HCWs at all levels.

A poor level of knowledge has been implicated in the rapid spread of infections in health facilities [9,10] and delay of treatment [11], which may put patients’ lives at risk. On the other hand, HCWs are not only at the forefront of the fight against this highly contagious infectious disease, but are also directly or indirectly affected by it, and the likelihood of acquiring this disease is higher among HCWs compared to the general population [12]. Therefore, it is crucial that HCWs across Ethiopia and in the Somali Region have adequate knowledge about all aspects of the disease, including clinical manifestation, diagnosis, proposed treatment, and established prevention strategies.

Thus, this study aimed to investigate the knowledge, attitudes, and practices of HCWs regarding COVID-19 infection. The findings may be useful in recommending any remedial measures and additional interventions in the study area to improve awareness, attitudes, and practices among HCWs.

## Methods

### Study Design, Setting, and Period

An institution-based cross-sectional study was conducted from June to August 2020 at Jigjiga University Teaching Hospital and two health centers in Jigjiga City, Somali Region, State of Ethiopia. There are two hospitals in the town; one is a multidisciplinary specialized teaching hospital with 351 inpatient beds and the other one is a general hospital with 193 inpatient beds. Currently, they provide health services to more than one million inhabitants in the catchment area. During data collection, Karamara General Hospital was turned into a COVID-19 treatment center and was not accessible for the study. Most of its staff were reassigned to other health facilities to support the provision of basic health services.

### Sample Size Determination and Sampling

Employees that were invited to participate in this study were all HCWs employed by the public health facilities in Jigjiga City. We included HCWs who were believed to have patient care or specimen contact, such as physicians, nurses, midwives, health officers, laboratory professionals, x-ray professionals, pharmacists and druggists, anesthetists, and biomedical professionals.

All public health facilities in the city, except for the one fully dedicated to COVID-19, were included. We contacted the human resources units to receive the lists of HCWs who have direct patient and specimen contact in the respective facilities. Finally, we came up with a total of 686 HCWs, which constituted the final sample size. 

### Eligibility Criteria

Only full-time employees (ie, HCWs) who were potentially at high risk of COVID-19 infection (ie, physicians, medical laboratory technologists, nurses, and midwives), were available during the data collection period, and were ready to take part in the study were included.

### Data Collection and Quality Control

A structured self-administered questionnaire was used to collect the data. The questionnaire was designed in reference to the study by Asaad et al regarding knowledge and attitudes toward the Middle East respiratory syndrome coronavirus, and it was adapted from the current interim guidance and information for HCWs published by the US Centers for Disease Control and Prevention (CDC) [5,13]. The questionnaire consists of six sociodemographic questions and 35 questions on knowledge, attitudes, and infection control practices related to COVID-19 in the health care setting. A pretest was done among 5.0% (34/686) of the study participants to estimate the duration required to complete the survey, ensure clarity of the questions, avoid potential bias, and validate the internal consistency of the items in attitude measurement. The Cronbach α value was .72, which was acceptable. The questionnaire has a satisfactory level of construct validity and internal consistency, according to this result.

The questionnaires were collected daily and checked for completeness; in the case of an incomplete questionnaire, the respondent was contacted in order to complete it. In addition, timely supervision of the data collection process was done by the investigators. During collection of the questionnaires, precautionary measures, such as wearing of masks and physical distancing, were observed. Data quality was ensured during data collection, and regular supervision and follow-up were performed.

### Measurements of Knowledge, Attitudes, and Practices

Knowledge questions were scored as 1 or 0 for correct or incorrect responses, respectively. The total knowledge score ranged from 0 (with no correct answer) to 15 (for all correct answers); a mean score of ≤13.7 indicated poor knowledge, and a mean score of >13.7 indicated good knowledge. There were 14 practice questions, which were scored on a 3-point scale from 1 (“always”) to 3 (“never”). Total practice scores ranged from 14 to 42; a mean score of >18.8 (answering “never” or “occasionally”) indicated poor practices, and a score of ≤18.8 (answering “always”) indicated good practices. Thus, the lower the practice scores were, the higher the probability of good practices, and vice versa. Attitude questions were scored on a 5-point Likert scale as follows: 1 (“strongly agree”), 2 (“agree”), 3 (“neutral”), 4 (“disagree”), and 5 (“strongly disagree”). There were six attitude questions. The total score ranged from 6 to 30; a mean score of >10.5 (answering “disagree,” “strongly disagree,” or “neutral”) indicated a negative attitude, and a score of ≤10.5 (answering “strongly agree” or “agree”) indicated a positive attitude. Therefore, the lower the attitude scores were, the higher the probability of positive attitudes, and vice versa.

### Data Analysis

Data were coded and entered into Epi Info software (version 3.5.1; CDC) and exported into Stata software (version 14.1; StataCorp LP) for analysis. Summary statistics, such as frequencies and proportions, were computed as appropriate. We conducted *t* tests and analyses of variance to analyze the relationship between the dependent (ie, knowledge, attitudes, and practices) and independent variables (ie, demographic characteristics of the participants). Spearman correlation was used to assess the relationship between mean knowledge and attitude scores. The differences in estimated variables were considered statistically significant at *P*<.05.

### Ethical Considerations

Ethical clearance and support letters were obtained from the Ethical Review Committee of the College of Medicine and Health Sciences, Jigjiga University. The support letter was then submitted to the public health facilities. Permission was then obtained from the health facilities’ director and department or section heads. Study participants were informed about the purpose and importance of the study through written informed consent before the data collection process. In addition, participants who were unwilling to take part in the study and those who needed to quit their participation at any stage were informed that they could do so without any restriction.

## Results

### Sociodemographic Characteristics

Of the 686 HCWs approached, a total of 434 responded (63.3% response rate). The mean age of the participants was 27.6 (SD 5.3) years, and the majority of the participants were male (293/434, 67.5%) and below 40 years of age (423/434, 97.5%). The majority of the participants were working in the referral hospital (345/434, 79.5%), were nurses (322/434, 74.2%), and had more than 5 years of experience (307/434, 70.7%). The main sources of information about COVID-19 were the WHO followed by government health authorities and media (Table 1).

**Table 1:** Sociodemographic characteristics of health care workers in Jigjiga City.

Variable			Value (N=434)
**Sex, n (%)**			
	Male	293 (67.5)	
	Female	141 (32.5)	
**Age in years**			
	Mean, SD	27.6 (5.3)	
	18-39, n (%)	423 (97.5)	
	≥40, n (%)	11 (2.5)	
**Occupation, n (%)**			
	Nurse (including midwives)	322 (74.2)	
	Physician	36 (8.3)	
	Pharmacist	26 (6.0)	
	Dentist	7 (1.6)	
	Laboratory technologist	31 (7.1)	
	X-ray physician	12 (2.8)	
**Place of work, n (%)**			
	Referral hospital	345 (79.5)	
	Karamara General Hospital	33 (7.6)	
	Ablele Health Center	44 (10.1)	
	Ayrdage Health Center	12 (2.8)	
**Work experience in years**			
	Mean, SD	8.3 (9.1)	
	0-4, n (%)	127 (29.3)	
	≥5, n (%)	307 (70.7)	
**Source of COVID-19 information, n (%)**			
	World Health Organization	241 (55.5)	
	Government site and media	105 (24.2)	
	Social media^a^	52 (12.0)	
	Other news media	30 (6.9)	
	Others	6 (1.4)	

^a^Social media platforms include WhatsApp, Facebook, and Telegram.

### Knowledge

Table 2 shows the details of the responses given by the health professionals for each knowledge question dealing with COVID-19 signs and symptoms, potential admission criteria required to identify patients at risk, approaches to prevent transmission in hospitals, and possible supportive treatment for patients with COVID-19.

Of the total HCWs who participated in the study, 73.3% (318/434) had sufficient knowledge (Table 3). Almost all HCWs were able to correctly identify COVID-19 key symptoms. Data from this study revealed that 95.4% (414/434) of respondents fully understood the common signs and symptoms of COVID-19. A total of 89.9% (390/434) were also aware of factors likely to be associated with severity of the disease.

The majority of the respondents understood the dynamics of COVID-19 infectiousness: 91.5% (397/434) of respondents were aware of the possibility that one could infect others before the onset of symptoms and 90.8% (394/434) of HCWs responded “true” to the question about droplets as a major transmission route. A considerable proportion of respondents (404/434, 93.1%) knew that the incubation period is not constant and could vary from 2 to 14 days.

**Table 2:** Health care workers’ (HCWs) knowledge regarding COVID-19.

Statements about knowledge	Responses by HCWs (N=434), n (%)		
	True	False	I don’t know
The common symptoms of COVID-19 are fever, fatigue, and dry cough.	414 (95.4)	13 (3.0)	7 (1.6)
The causative agent of COVID-19 is coronavirus.	410 (94.4)	12 (2.8)	12 (2.8)
The incubation period of COVID-19 is 2 to 14 days.	404 (93.1)	21 (4.8)	9 (2.1)
Not all persons with COVID-19 will develop severe cases. Only those who are elderly and have chronic illnesses are likely to be severe cases.	390 (89.9)	33 (7.6)	11 (2.5)
COVID-19 can be transmitted through direct contact of respiratory droplets when infected persons cough or sneeze.	394 (90.8)	28 (6.4)	12 (2.8)
The disease can be transmitted from asymptomatic patients.	397 (91.5)	26 (6.0)	11 (2.5)
Training and observation of standard precautionary measures are required by caregiving personnel in suspected and probable cases of COVID-19 infection.	392 (90.3)	30 (6.9)	12 (2.8)
Visitors to patients with suspected, probable, and confirmed cases of COVID-19 infection should be limited, both in hospital and at home.	406 (93.6)	22 (5.1)	6 (1.3)
The disease can be spread by people touching a surface or object that has the virus on it and then touching their own mouth or nose or possibly their eyes.	401 (92.4)	22 (5.1)	11 (2.5)
Isolation and treatment of people who are infected with the COVID-19 virus are effective ways to reduce the spread of the virus.	404 (93.1)	23 (5.3)	7 (1.6)
People who have contact with someone infected with the COVID-19 virus should be immediately isolated in a proper place. In general, the observation period is 14 days.	400 (92.2)	24 (5.5)	10 (2.3)
A person with mild symptoms of COVID-19 must remain at home until resolution of clinical symptoms.	398 (91.7)	30 (6.9)	6 (1.4)
Standard precautions should be followed by health care providers in dealing with suspected, probable, and confirmed cases of COVID-19 infection.	395 (91.0)	29 (6.7)	10 (2.3)
Oxygen therapy should be given to all cases of severe COVID-19 with acute respiratory infection.	369 (85.0)	54 (12.5)	11 (2.5)
Ventilation with an endotracheal tube must be carried out in patients with confirmed and/or suspected COVID-19 with clinical manifestations of acute respiratory distress symptoms.	379 (87.3)	40 (9.2)	15 (3.5)

**Table 3:** Knowledge, attitude, and practice levels among health care workers.

Domain	Good level (N=434), n (%)	Poor level (N=434), n (%)
Knowledge	318 (73.3)	116 (26.7)
Attitude	238 (54.8)	196 (45.2)
Practice	267 (61.5)	167 (38.5)

With respect to prevention of transmission from known or suspected patients, HCWs knew most of the preventive measures. The majority of HCWs (400/434, 92.2%) responded that isolation could be one of the possible ways to prevent COVID-19. The majority of HCWs (395/434, 91.0%) responded that standard precautions should be followed by health care providers when dealing with suspected, probable, and confirmed cases of COVID-19 infection (Table 2).

### Attitude

Of the total of HCWs who participated in the study, 54.8% (238/434) had positive attitudes (Table 3). Only 48.8% (212/434) were conﬁdent that COVID-19 can be controlled by public health institutes. More than half of the HCWs (226/434, 52.1%) agreed that this disease can be prevented, and less than half of the HCWs (214/434, 49.3%) agreed that it is imperative to use surgical masks when working with patients with COVID-19; unfortunately, 52.1% (226/434) of the HCWs were not confident in dealing with patients with COVID-19 (Table 4).

**Table 4:** Health care workers’ (HCWs) attitudes toward COVID-19.

Statements about attitudes	Responses by HCWs (N=434), n (%)				
	Strongly agree	Agree	Neutral	Disagree	Strongly disagree
Caring for patients with COVID-19 infection may be a threat to health care personnel.	226 (52.1)	147 (33.9)	28 (6.4)	24 (5.5)	9 (2.1)
Public health agencies, like the Ethiopia Public Health Institute, can control the outbreak of COVID-19.	212 (48.8)	121 (27.9)	42 (9.7)	50 (11.5)	9 (2.1)
COVID-19 can have a negative effect on the economies of the countries involved.	231 (53.2)	135 (31.1)	24 (5.5)	36 (8.3)	8 (1.9)
It is important to report suspected cases to health authorities.	301 (69.3)	104 (24.0)	11 (2.5)	13 (3.0)	5 (1.2)
COVID-19 is preventable.	226 (52.1)	136 (31.3)	33 (7.6)	23 (5.3)	16 (3.7)
It is imperative to use surgical masks when working with patients with COVID-19.	214 (49.3)	136 (31.3)	23 (5.3)	26 (6.0)	35 (8.1)

### Practice

Of the 434 HCWs who participated in the study, 61.5% (n=267) practiced precautionary measures to prevent COVID-19 (Table 3). Practical measures put in place by HCWs in order to protect themselves and their families are presented in Table 5. A total of 72.4% (n=314) of the 434 HCWs reported that they always wore personal protective equipment when coming into contact with the patients, and 67.5% (n=293) washed their hands before and after touching each patient. Other measures observed included the following: 72.8% (n=316) of the HCWs always avoided going to crowded places and 71.4% (n=310) always avoided shaking hands, hugging, or kissing. In addition, 71.7% (n=311) of the HCWs always kept a safe distance and 71.0% (n=308) covered their mouths when sneezing or coughing. Unfortunately, as high as 72.6% (n=315) of the participants had avoided patients with symptoms suggestive of COVID-19.

**Table 5:** Health care workers’ (HCWs) practices regarding COVID-19.

Statements about practices	Responses by HCWs (N=434), n (%)		
	Always	Occasionally	Never
I always wear the personal protective equipment (PPE) when handling a patient.	314 (72.4)	101 (23.3)	19 (4.4)
I wash my hands with water and soap before putting on the gloves.	302 (69.6)	105 (24.2)	27 (6.2)
For residual protection, I use hand sanitizer after washing my hands with soap and water.	303 (69.8)	103 (23.7)	26 (6.5)
I wash my hands between patients (before and after examining).	293 (67.5)	105 (24.2)	36 (8.3)
I wash my hands whenever they are soiled.	298 (68.7)	113 (26.0)	23 (5.3)
I wash my hands after blowing my nose or covering a sneeze.	289 (66.6)	114 (26.3)	31 (7.1)
I wash my hands with soap and water and apply hand sanitizer when leaving the health facility.	208 (71.0)	107 (24.6)	19 (4.4)
When I sneeze or cough, I cover my nose or mouth with a tissue or clean cloth or do so into my elbow.	308 (71.0)	106 (24.4)	20 (4.6)
I keep at least 1 meter of distance from a sick person.	311 (71.7)	108 (24.9)	15 (3.4)
I avoid shaking hands with, hugging, or kissing colleagues and patients.	310 (71.4)	110 (25.4)	14 (3.2)
I avoid touching my mouth, nose, and eyes with my hands if I have not washed them with water and soap.	307 (70.7)	117 (27.0)	10 (2.3)
I avoid going to crowded places.	316 (72.8)	107 (24.7)	11 (2.5)
I safely dispose of the used PPE items when I finish the service.	315 (72.6)	100 (23.0)	19 (4.4)
I avoid patients with signs and symptoms suggestive of COVID-19.	315 (72.6)	91 (21.0)	28 (6.4)

### Distribution of Knowledge, Attitudes, and Practices

The relationships between sociodemographic characteristics and knowledge, attitudes, and practices regarding COVID-19 are demonstrated in Table 6. The mean knowledge, attitude, and practice scores were 13.7 (SD 2.6), 10.5 (SD 4.1), and 18.8 (SD 5.8), respectively. Male participants had a higher mean knowledge score than female participants (13.9, SD 2.3, and 13.3, SD 3.1, respectively; *P*=.01). Nurses had the highest knowledge scores, followed by pharmacists and other professions (*P*=.04). No difference in knowledge was found with respect to age and work experience. Mean knowledge scores were significantly related to the place of work as well as to the source of knowledge. Participants from the referral hospital had a significantly higher mean knowledge score compared to those working in other health facilities (*P*<.001). Additionally, significantly increased knowledge scores were observed in those getting information from WHO websites followed by government media (*P*<.001).

Similarly, male participants had more positive attitudes than female participants (10.3 , SD 4.1, and 10.8, SD 4.1, respectively) with no statistically significant difference. No difference in attitude was found with respect to qualification, work experience, and age. A positive attitude was significantly related to the place of work as well as to the source of knowledge. Participants from the referral hospital had a significantly lower mean attitude score compared to those working in other health facilities (*P*<.001). Additionally, a significantly decreased attitude score was detected among those getting information from social media, followed by WHO websites (*P*=.02).

Likewise, male participants tended to practice precautionary measures more than female participants (18.6, SD 5.8, and 19.3, SD 6.3, respectively) with no statistically significant difference. No difference in practice was found with respect to qualification and work experience. Good practice was significantly related to age, place of work, and source of knowledge. Older participants (≥40 years) tended to practice precautionary measures more than those in younger age groups (*P*=.04). Additionally, significantly better practices were observed among participants working at Ayrdage Health Center (*P*=.01) and those getting information from government sites and media (*P*=.02).

**Table 6:** Distribution of knowledge, attitude, and practice scores among health care workers.

Variable			Knowledge score^a^, mean (SD)		*t* test^b^ (*df*=432)		*F* test^c^ (*df*=433)		*P* value		Attitude score^d^, mean (SD)		*t* test (*df*=432)		*F* test (*df*=433)	*P* value		Practice score^e^, mean (SD)		*t* test (*df*=432)			*F* test (*df*=433)	*P* value	
Overall			13.7 (2.6)		—^f^		—		—		10.5 (4.1)		—		—	—		18.8 (5.8)		—			—	—	
**Sex**																									
	Male	13.9 (2.3)		2.49		—		.01		10.3 (4.1)		–1.02		—			.31		18.6 (5.8)		–1.18	—			.24
	Female	13.3 (3.1)		—		—		—		10.8 (4.1)		—		—			—		19.3 (6.3)		—	—			—
**Age in years**																									
	18-39	13.7 (2.6)		0.014		—		.99		10.5 (4.1)		0.24		—			.81		18.9 (6.0)		2.01	—			.04
	≥40	13.7 (1.8)		—		—		—		10.2 (2.9)		—		—			—		15.3 (2.7)		—	—			—
**Occupation**																									
	Nurse (including midwives)	13.9 (2.2)		—		2.39		.04		10.3 (3.8)		—		1.45			.21		19.2 (6.1)		—	1.61			.16
	Physician	12.9 (3.9)		—		—		—		11.0 (4.4)		—		—			—		19.3 (5.9)		—	—			—
	Pharmacist	13.2 (2.4)		—		—		—		10.7 (3.8)		—		—			—		16.5 (3.5)		—	—			—
	Dentist	12.2 (3.5)		—		—		—		9.9 (2.3)		—		—			—		18.3 (4.5)		—	—			—
	Laboratory technologist	13.0 (3.5)		—		—		—		10.9 (5.6)		—		—			—		17.3 (5.9)		—	—			—
	X-ray physician	13.6 (3.1)		—		—		—		13.2 (6.7)		—		—			—		17.4 (4.4)		—	—			—
**Place of work**																									
	Referral hospital	14.3 (1.8)		—		40.5		<.001		10.1 (4.1)		—		5.93			<.001		18.8 (6.0)		—	3.87			.01
	Karamara General Hospital	12.3 (3.4)		—		—		—		12.0 (4.2)		—		—			—		17.6 (4.4)		—	—			—
	Ablele Health Center	10.6 (4.0)		—		—		—		12.3 (3.8)		—		—			—		20.9 (6.6)		—	—			—
	Ayrdage Health Center	12.6 (2.4)		—		—		—		11.5 (2.4)		—		—			—		15.3 (1.8)		—	—			—
**Work experience in years**																									
	0-4	13.8 (2.3)		0.41		—		.68		9.9 (3.4)		–1.78		—			.08		18.9 (6.0)		0.54	—			.59
	≥5	13.7 (2.7)		—		—		—		10.7 (4.4)		—		—			—		15.3 (2.7)		—	—			—
**Source of COVID-19 information**																									
	World Health Organization	14.0 (2.0)		—		5.94		<.001		10.4 (3.9)		—		2.96			.02		19.2 (6.1)		—	2.88			.02
	Government site and media	13.9 (2.2)		—		—		—		11.1 (4.4)		—		—			—		17.2 (4.5)		—	—			—
	Social media	13.3 (3.1)		—		—		—		9.1 (3.3)		—		—			—		19.1 (6.1)		—	—			—
	Other news media	12.6 (3.9)		—		—		—		11.6 (4.4)		—		—			—		19.7 (7.0)		—	—			—
	Other	9.8 (6.9)		—		—		—		12.2 (7.0)		—		—			—		22.2 (11.2)		—	—			—

^a^Knowledge questions were scored as 1 or 0 for correct or incorrect responses, respectively. The total knowledge score ranged from 0 (with no correct answer) to 15 (for all correct answers).

^b^The *t* test was used to compare two mean scores within demographic characteristic categories with two subitems (eg, sex) for knowledge, attitude, and practice.

^c^The analysis of variance (ie, *F* test) was used to compare three or more mean scores within demographic characteristic categories with three or more subitems (eg, occupation) for knowledge, attitude, and practice.

^d^Attitude questions were scored on a 5-point Likert scale from 1 (“strongly agree”) to 5 (“strongly disagree”). The total score ranged from 6 to 30.

^e^Practice questions were scored on a 3-point scale from 1 (“always”) to 3 (“never”). Total practice scores ranged from 14 to 42.

^f^Values are reported in the top row of each category only when the corresponding statistical test (ie, *t* test or *F* test) was conducted.

### Correlation Between Knowledge, Attitude, and Practice Domains

Table 7 presents the correlation coefficients between the domains of knowledge, attitude, and practice. It was found that the attitude (*r*=–0.295, *P*<.001) and practice (*r*=–0.298, *P*<.001) domains were inversely associated with the knowledge score. A significant positive correlation was found between attitude and practice (*r*=0.173, *P*<.001). The lower the attitude and practice scores were, the higher the probability of positive attitudes and good practices, while the higher the knowledge scores were, the higher the probability of good knowledge. Therefore, good knowledge of COVID-19 was directly associated with a positive attitude and good practices. Similarly, a positive attitude was directly associated with good practices.

**Table 7:** Correlation analysis (Pearson *r* and two-tailed *P* value) among the research variables.

Variable		Knowledge	Attitude	Practice
**Knowledge**				
	*r*	1	–0.295^a^	–0.298
	*P* value	—^b^	<.001	<.001
**Attitude**				
	*r*	–0.295	1	0.173
	*P* value	<.001	—	<.001
**Practice**				
	*r*	–0.298	0.173	1
	*P* value	<.001	<.001	—

^a^The correlation is significant at a significance level of .05 (two-tailed).

^b^Not applicable.

### Availability of Data and Materials

The data set for this study is available from the corresponding author on reasonable request.

## Discussion

### Principal Findings

This study assessed the knowledge, attitudes, and practices related to COVID-19 among HCWs working at public health facilities. In this study, responses from 434 HCWs regarding sociodemographic characteristics, knowledge, attitudes, and infection prevention practices were analyzed. We found that the majority of HCWs had good knowledge and that over half of them had positive attitudes and good practices regarding COVID-19.

Our study found that most of the HCWs were well informed about COVID-19–related knowledge during the outbreak. Among these HCWs, the level of knowledge about COVID-19 was higher among male participants and nurses. From our study, we found that 73.3% (318/434) of the HCWs had sufficient knowledge about COVID-19, which is almost the same as the values reported by studies conducted in Uganda and Northern Ethiopia [14,15], where 69% and 74% of HCWs had sufficient knowledge, respectively. The proportion of HCWs with sufficient knowledge in this study was lower than in studies conducted in Vietnam and China [16,17].

This study explored the overall mean knowledge score, which was 13.7 (SD 2.6). These results were higher compared to a study from Vietnam that reported a mean knowledge score of 8.17 (SD 1.3). Their findings showed that HCWs had a high level of knowledge and a positive attitude regarding the COVID-19 outbreak [16]. In contrast, our results showed a lower knowledge level and a less positive attitude than in studies conducted in Egypt [18].

In general, having sufficient knowledge may reflect the successful dissemination of information about COVID-19 by different media. In this regard, most of the participants in this study used information from international and governmental media (ie, websites and veriﬁed social media pages). Our study suggests that the majority of HCWs used WHO websites as a source of information for COVID-19. This could be explained by the fact that the WHO website is regularly updated with facts. This suggests that such media should be frequently used to disseminate information on COVID-19 by the stakeholders involved in the COVID-19 response.

About 52.1% (226/434) of HCWs believed that caring for patients with COVID-19 may be a threat to HCWs, which is similar to findings by a study conducted in China [17] that showed that around 85% of the surveyed HCWs were afraid of getting infected while caring for their patients. HCWs help patients during their routine tasks, such as patient consultation, infusion, dressing changes, and surgery. They must also deal with many other emergency situations, and they may become infected with the virus if they are not cautious. Similarly, about 14.1% (61/434) of HCWs believed that wearing general medical masks may not protect the spread COVID-19, contrary to ﬁndings by Ng et al, which showed adequate protection [19].

Our study revealed that more than two-fifths of HCWs had negative attitudes toward COVID-19, which is in congruence with a knowledge, attitude, and practice study on COVID-19 in Uganda among HCWs [15], but in contrast to studies about COVID-19 in China and Ethiopia [17,20]. This study also explored the overall mean attitude score, which was 10.5 (SD 4.1). These results were lower than those in a study from Egypt that reported a mean knowledge score of 13.3 (SD 2.1) [18].

However, a Spearman analysis found a significant negative correlation between the mean knowledge and attitude scores of HCWs regarding COVID-19 (*r*=–0.295, *P*<.001), which is consistent with studies conducted in Vietnam and Egypt [16,18]. In other words, the lower the attitude scores were, the higher the probability of positive attitudes, while the higher the knowledge scores were, the higher the probability of good knowledge and practice. Therefore, good knowledge of COVID-19 was directly associated with a positive attitude and good practices. Similarly, a good attitude was positively associated with good practices.

Our study showed that the majority of HCWs had good COVID-19 prevention practices, which is in line with ﬁndings by other studies on COVID-19 [14,15,17]. The majority of the HCWs were following key infection prevention and control practices recommended by the Ministry of Health Ethiopia and the WHO. These included regular hand hygiene, social distancing, and wearing of personal protective equipment when in high-risk situations. A total of 72.4% (314/434) and 67.5% (293/434) of HCWs reported wearing personal protective equipment when in contact with patients as well as washing hands before and after handling patients, respectively. These are vital practices to prevent transfer of COVID-19 from patient to patient and to the HCWs themselves. This finding is in line with a study conducted in Ethiopia [20], but the values are lower than in many other studies [21-23]. However, up to 72.6% (315/434) of HCWs admitted having avoided patients with symptoms suggestive of COVID-19. This can be attributed to a global shortage of personal protective equipment [24,25].

It was also observed that occupation was significantly associated with knowledge. Nurses and pharmacists showed relatively better knowledge than other health professionals. This finding is in line with a study conducted in Vietnam that showed that pharmacists had better knowledge than other health professionals [16]. Similarly, a significant increase in the knowledge score was detected in those getting information from WHO websites, followed by government media. This finding corroborates a report from Egypt that found that HCWs who gained information about the disease from the internet, either through social media or the WHO website, had better knowledge scores [18]. This could be explained by the fact that younger, highly educated persons tend to use the internet more than older, less educated persons. In contrast, a study conducted in Uganda showed that HCWs who gained information from the traditional news media, such as television, radio, and newspapers, had more knowledge [15].

Our results also found that good knowledge was significantly associated with positive attitudes. This is in line with several studies that found an association between the COVID-19 knowledge level of HCWs and their attitudes [16,21]. Knowledge of HCWs is a very important prerequisite for positive attitudes and for promoting positive practices. Attitudes toward COVID-19 were also better among HCWs who got information from social media, followed by WHO websites. This is in line with a study that showed that social media exposure to COVID-19 information influences the adoption of preventive attitudes and behaviors through shaping risk perception [26]. Thus, understanding the role of social media during the pandemic could help policy makers and communicators to develop better communication strategies that enable HCWs to adopt appropriate attitudes and behaviors.

We also observed that older participants tended to practice precautionary measures more than younger participants; our findings support a report from Bangladesh that showed that a notable proportion of young adults did not have good preventive practices regarding COVID-19 [27]. This could be explained by the fact that younger participants considered themselves less at risk compared to older participants.

### Limitations

Our study has some limitations. Firstly, no standardized tool for assessing knowledge, attitudes, and practices regarding COVID-19 has been previously validated. We adapted and modiﬁed a previously published tool for assessment of knowledge, attitudes, and practices regarding the prevention of respiratory tract infections. Secondly, only HCWs in public health facilities in parts of the Somali Region were surveyed, and the results of this study may not reﬂect the knowledge, attitudes, and practices of HCWs in the private sector. A similar study may be extended to the community.

### Conclusions

This study highlights that more than 73% of HCWs had sufficient knowledge on the transmission, diagnosis, and prevention of COVID-19; more than half had positive attitudes toward COVID-19; and two-thirds had good practices regarding COVID-19 precautionary measures. There was a statistically signiﬁcant difference in the level of knowledge about COVID-19 among HCWs. In a nutshell, the overall levels of knowledge and good practice were better compared to the positive attitude level. Hence, efforts to enhance the capacity of HCWs can be very helpful for the containment of the pandemic through enhancing positive attitudes and good practices.

## Abbreviations

CDC: Centers for Disease Control and Prevention
HCW: health care worker
WHO: World Health Organization
